# Supplementary material for: Cryo-electron microscopy structure of an archaeal ribonuclease P holoenzyme
Source: Nat Commun. 2019 Jun 13;10:2617. doi: 10.1038/s41467-019-10496-3 (PMC6565675; doi:10.1038/s41467-019-10496-3)
Supplement: Supplementary file 1 — Supplementary Information [file 41467_2019_10496_MOESM1_ESM.pdf]

Supplementary Information

Cryo-electron microscopy structure of an archaeal ribonuclease P holoenzyme

Wan et al.

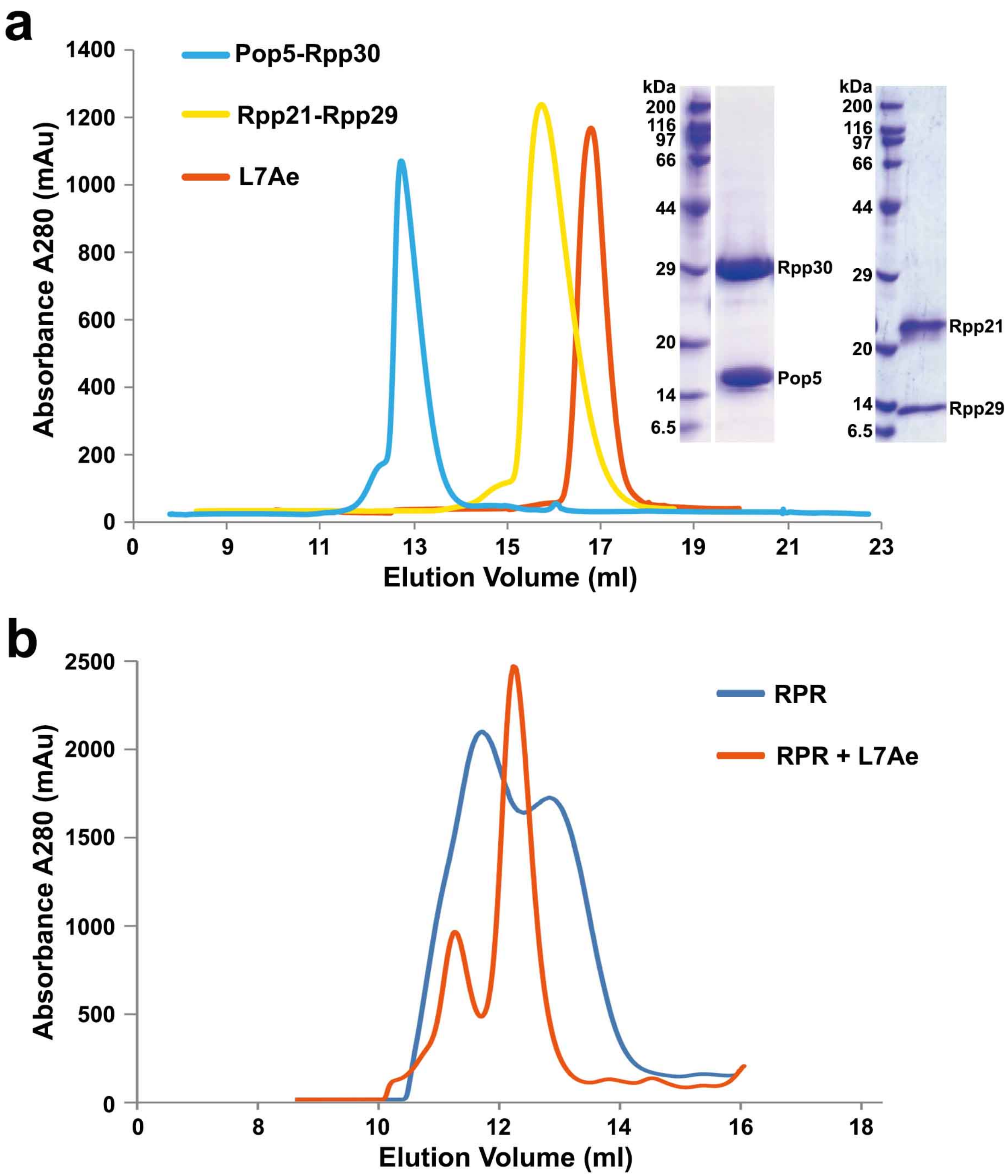

Supplementary Figure 1

Supplementary Fig. 1 Protein purification and RPR transcription. **(a)** SEC profiles of L7Ae, the $(Pop5\text{-}Rpp30)_2$ and Rpp29-Rpp21 subcomplexes. Coomassie-stained SDS-PAGE gels of the corresponding peaks in the profiles are also shown. **(b)** SEC profiles of *in vitro* transcribed RPR with or without L7Ae.

a

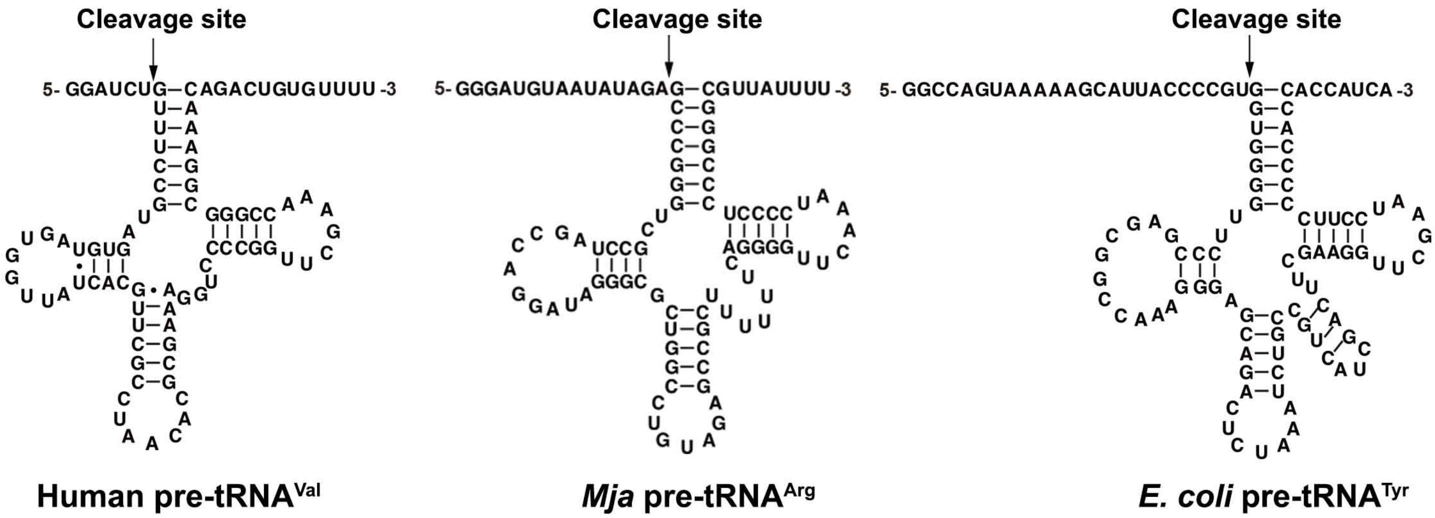

b

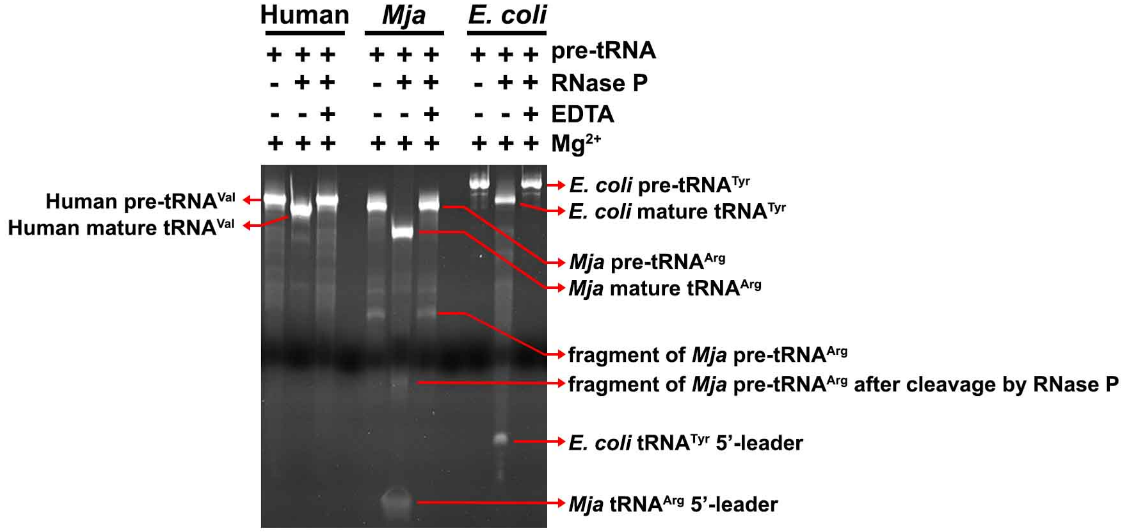

Supplementary Figure 2

Supplementary Fig. 2 tRNA processing activity assay. **(a)** Secondary structures of human pre-tRNA^{Val} (left), *M. jannaschii* pre-tRNA^{Arg} (middle) and *E. coli* pre-tRNA^{Tyr} (right). **(b)** *In vitro* tRNA processing assay of the *Mj*aRNase P holoenzyme with different pre-tRNA substrates shown in (a). Cleaved 5'-leaders of *E. coli* pre-tRNA^{Tyr} and *M. jannaschii* pre-tRNA^{Arg} are denoted at the bottom of the gel. The cleaved 5'-leader of human pre-tRNA^{Val} is too short (six nucleotides) to be seen in the gel. It is likely that during the preparation of pre-tRNA^{Arg}, a fragment of pre-tRNA^{Arg} that contains the 5'-leader was generated and after cleavage by RNase P, this fragment gave rise to an extra band that is not present without RNase P or with EDTA.

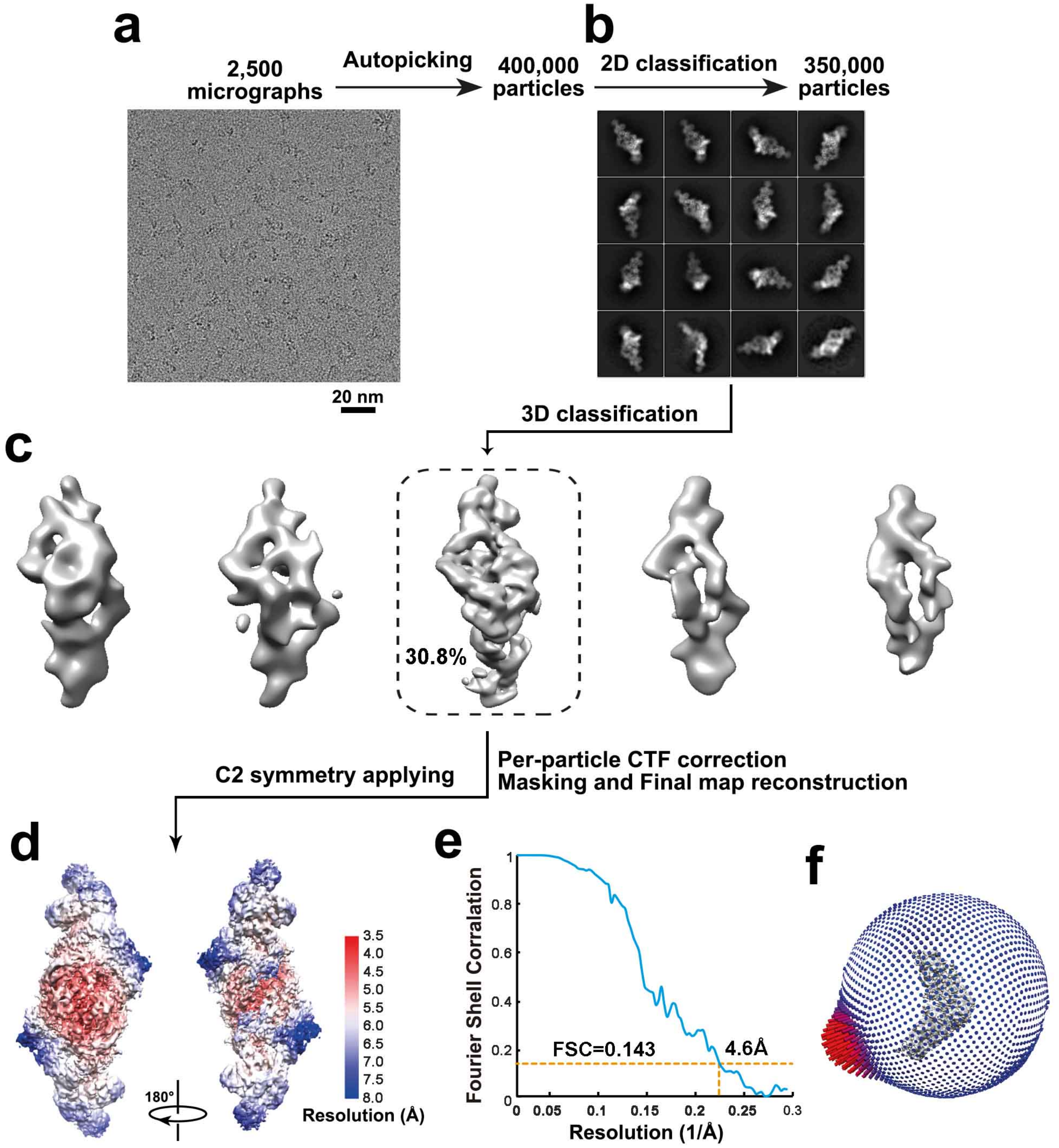

Supplementary Figure 3

Supplementary Fig. 3 Cryo-EM analyses of the *Mja*RNase P holoenzyme. **(a)** A representative cryo-EM micrograph of the *Mja*RNase P holoenzyme. **(b)** Selected reference-free 2D class averages of the *Mja*RNase P particles. **(c)** 3D classes of the cryo-EM particles used for *Mja*RNase P reconstruction. **(d)** Local resolution estimation of the *Mja*RNase P complex. **(e)** Gold standard Fourier shell correlation (FSC) curve of the final reconstructed EM map of the *Mja*RNase P holoenzyme. **(f)** Angular distribution of the particles used for the final reconstruction of the *Mja*RNase P holoenzyme.

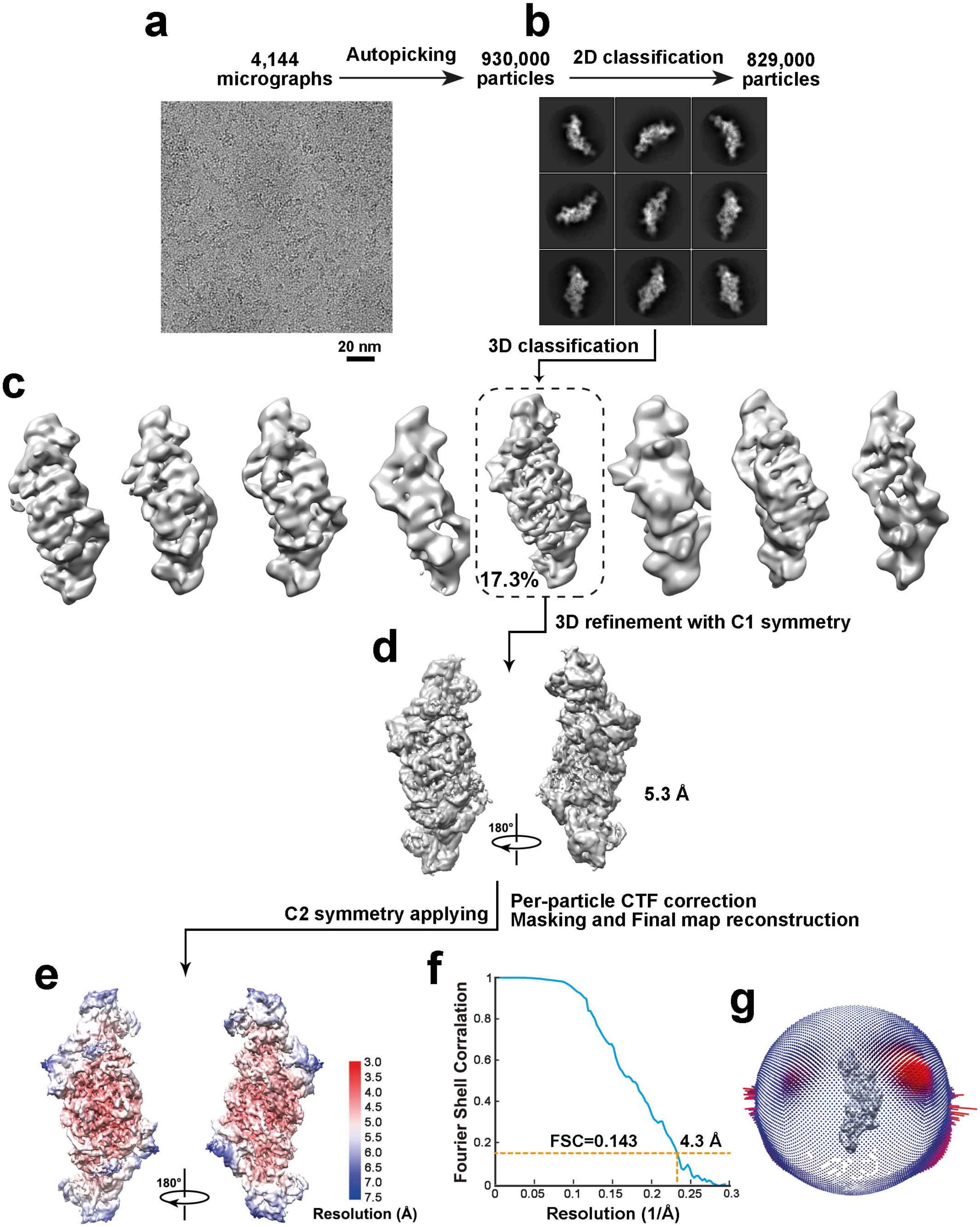

Supplementary Figure 4

Supplementary Fig. 4 Cryo-EM analyses of *Mja*RNase P in complex with *E.coli* $tRNA^{Tyr}$. **(a)** A representative cryo-EM micrograph of the *Mja*RNase P-tRNA complex. **(b)** Selected reference-free 2D class averages of *Mja*RNase P-tRNA complex particles. **(c)** 3D classes of the cryo-EM particles used for *Mja*RNase P-tRNA complex construction. **(d)** 3D refinement of *Mja*RNase P-tRNA complex using C1 symmetry. **(e)** Local resolution map of the *Mja*RNase P-tRNA complex. C2 symmetry was applied in this round of 3D refinement to improve the resolution. **(f)** Gold standard FSC curve of the final reconstructed EM map of *Mja*RNase P-tRNA complex. **(g)** Angular distribution of the particles used for the final reconstruction of the *Mja*RNase P-tRNA complex.

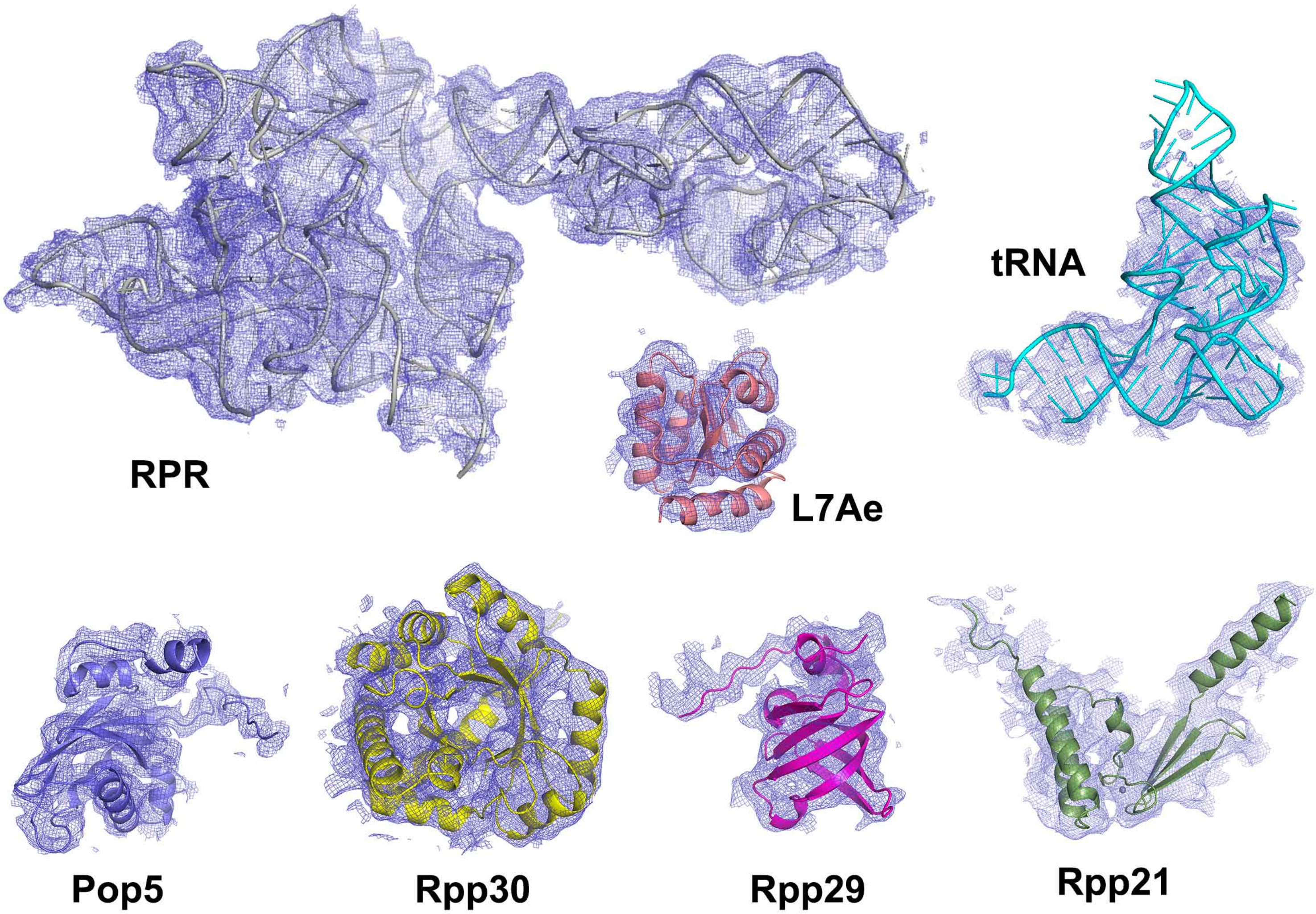

Supplementary Figure 5

Supplementary Fig. 5 Cryo-EM density maps of individual protein and RNA subunits in the *Mja*RNase P-pre-tRNA^{Tyr} complex. Cartoons of each structure are fit into the EM density map and colored as in Fig. 2a.

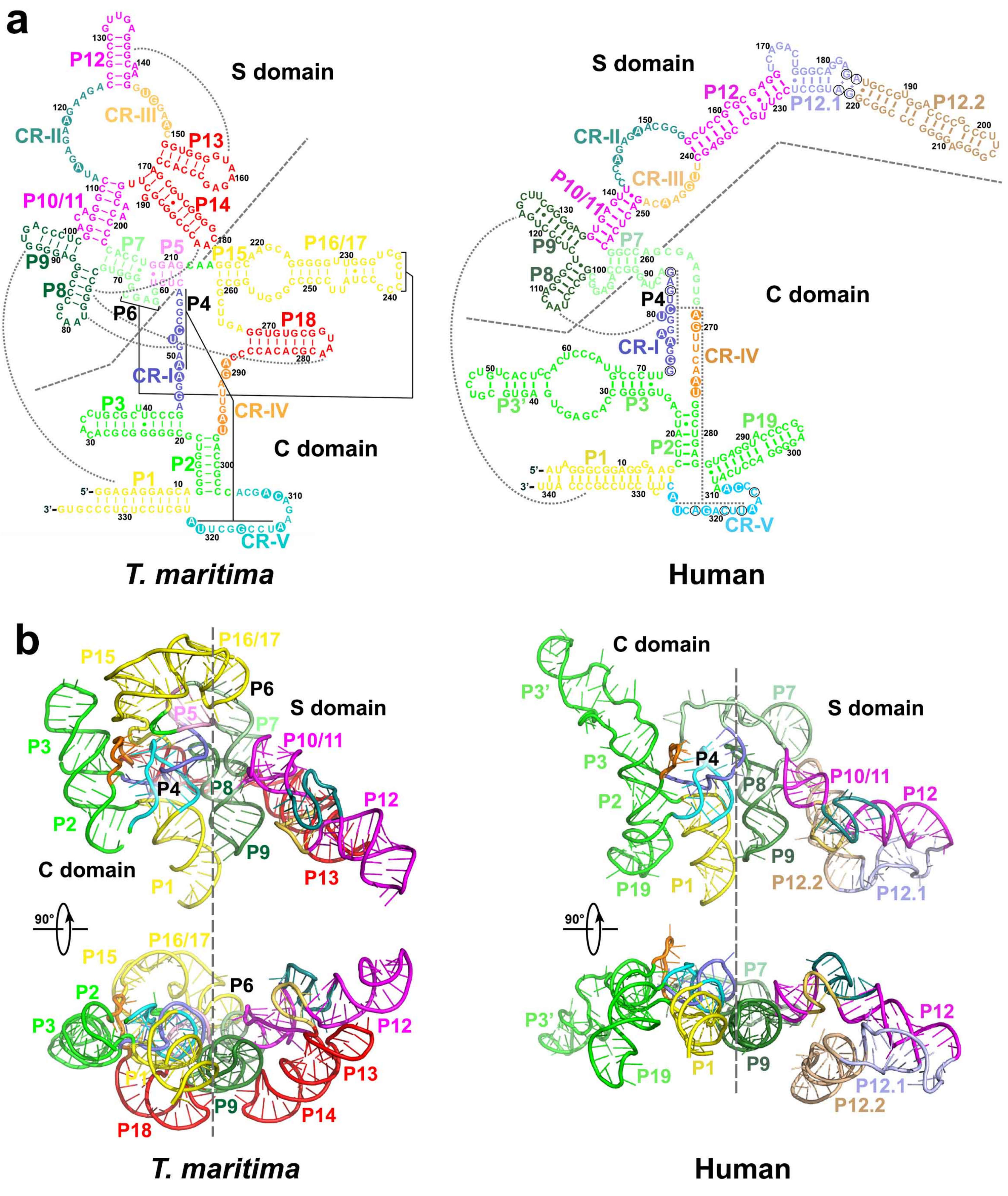

Supplementary Figure 6

Supplementary Fig. 6 RPR structures of *T. maritima* and human RNase P. **(a)** Secondary structures of *T. maritima* (left) and human (right) RNase P RPR. RNA stems are colored as in Fig. 3a. **(b)** Two orthogonal views of the overall structure of *T. maritima* (left) and human (right) RPR.

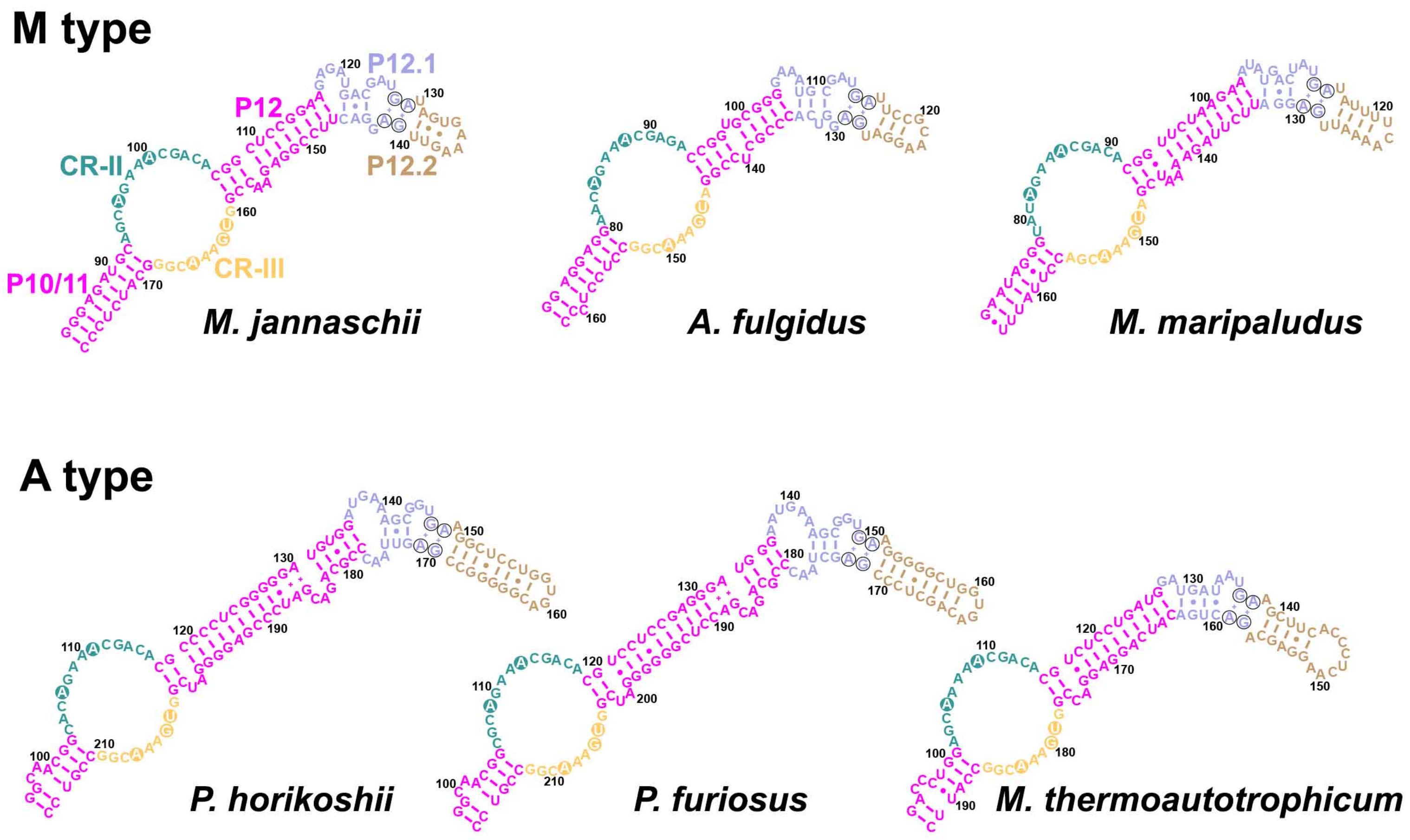

Supplementary Figure 7

Supplementary Fig. 7 Secondary structural models of the conserved K-turn in the S domain of RPR in representative archaeal organisms. The conserved regions are colored as in Fig. 3a. The conserved K-turn nucleotides are highlighted with open circles.

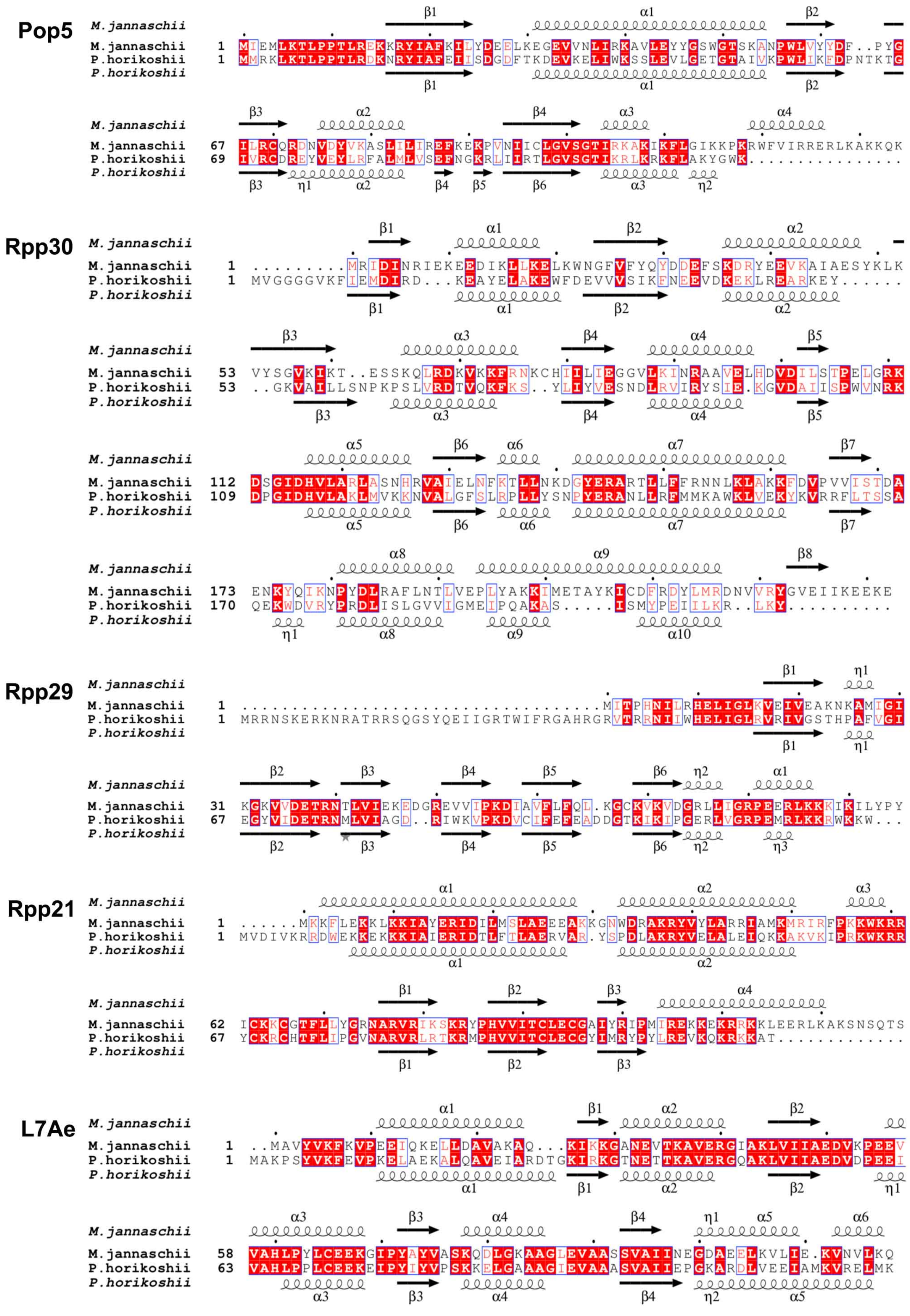

Supplementary Figure 8

Supplementary Fig. 8 Sequence alignment of RNase P RPRs of *M. jannaschii* and *P. horikoshii*. Conserved residues are colored in red. Secondary structural elements were assigned based on the cryo-EM structure of *Mja*RNase P reported in this study and the crystal structures of *P. horikoshii* RNase P protein components.

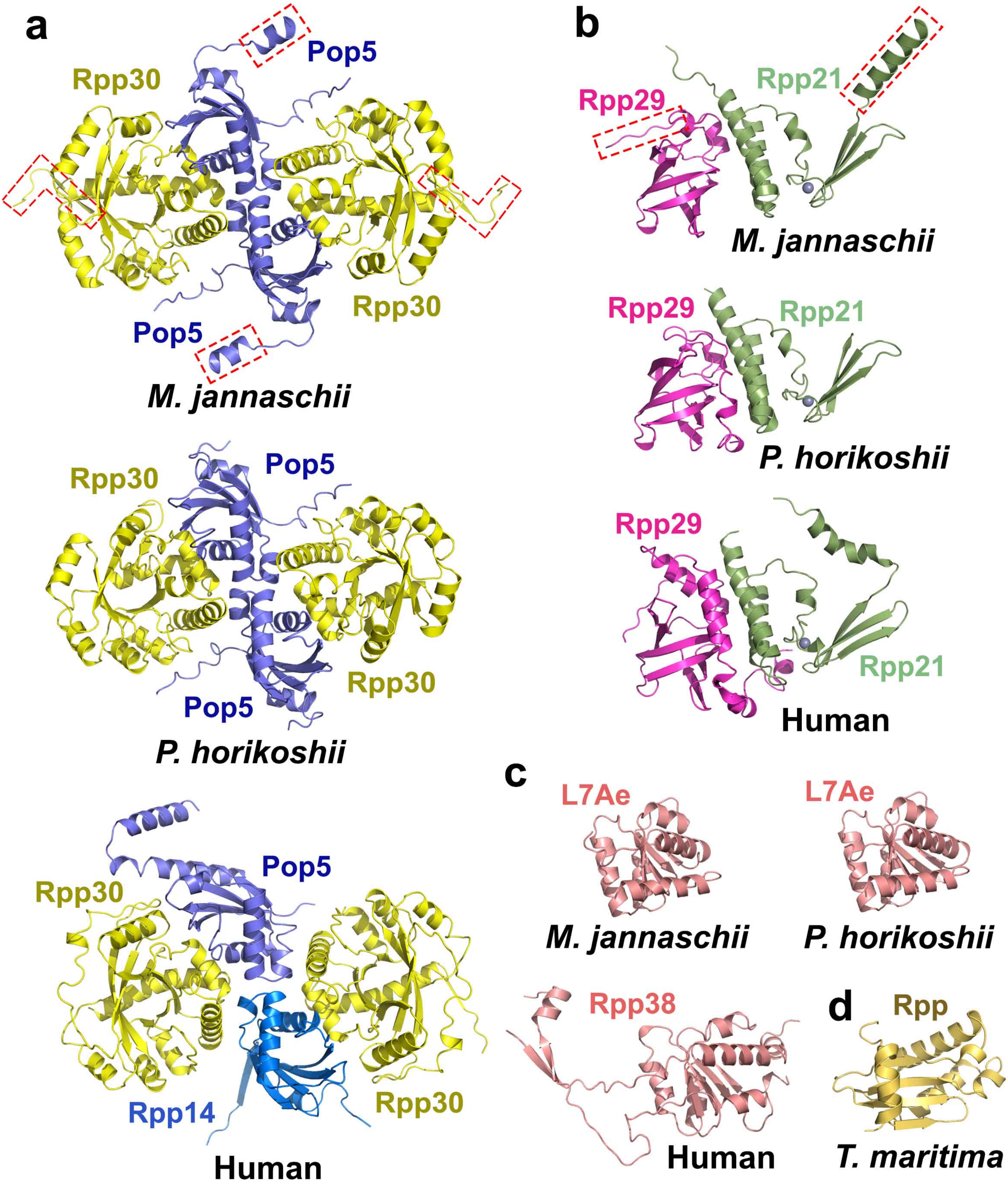

Supplementary Figure 9

Supplementary Fig. 9 Structure comparison of RNase P protein components. **(a)** Cartoon representation of *M. jannaschii* (Pop5-Rpp30)$_2$ (top), *P. horikoshii* (Pop5-Rpp30)$_2$ (middle) and human Pop5-(Rpp30)$_2$-Rpp14 (bottom). Extra structural elements of *M. jannaschii* Pop5 and Rpp30 compared with their *P. horikoshii* counterparts are highlighted by dashed red boxes. **(b)** Cartoon representation of *M. jannaschii* Rpp29-Rpp21 (top), *P. horikoshii* Rpp29-Rpp21 (middle), and human Rpp29-Rpp21 (bottom). Extra structural elements of *M. jannaschii* Rpp21 and Rpp29 compared with their *P. horikoshii* counterparts are highlighted by dashed red boxes. **(c)** Structure comparison of *M. jannaschii* L7Ae (left), *P. horikoshii* L7Ae (right) and human Rpp38 (bottom). **(d)** Cartoon representation of *T. maritima* Rpp.

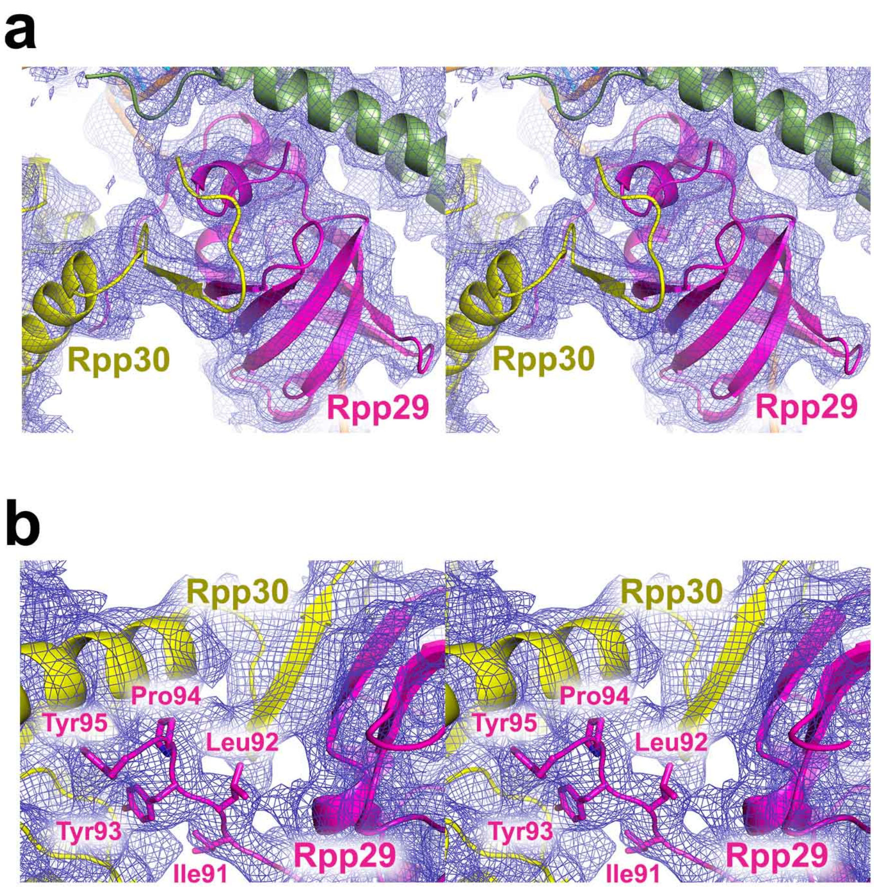

Supplementary Figure 10

Supplementary Fig. 10 Stereo view of electron density maps of the interfaces between Rpp30 and Rpp29. **(a)** Close-up view of the electron density map of the interface between the Rpp30 C terminal β strand and the β barrel of Rpp29. **(b)** Close-up view of the electron density map of the interface between the C-terminal tail of Rpp29 and the hydrophobic groove of Rpp30.

Rpp30

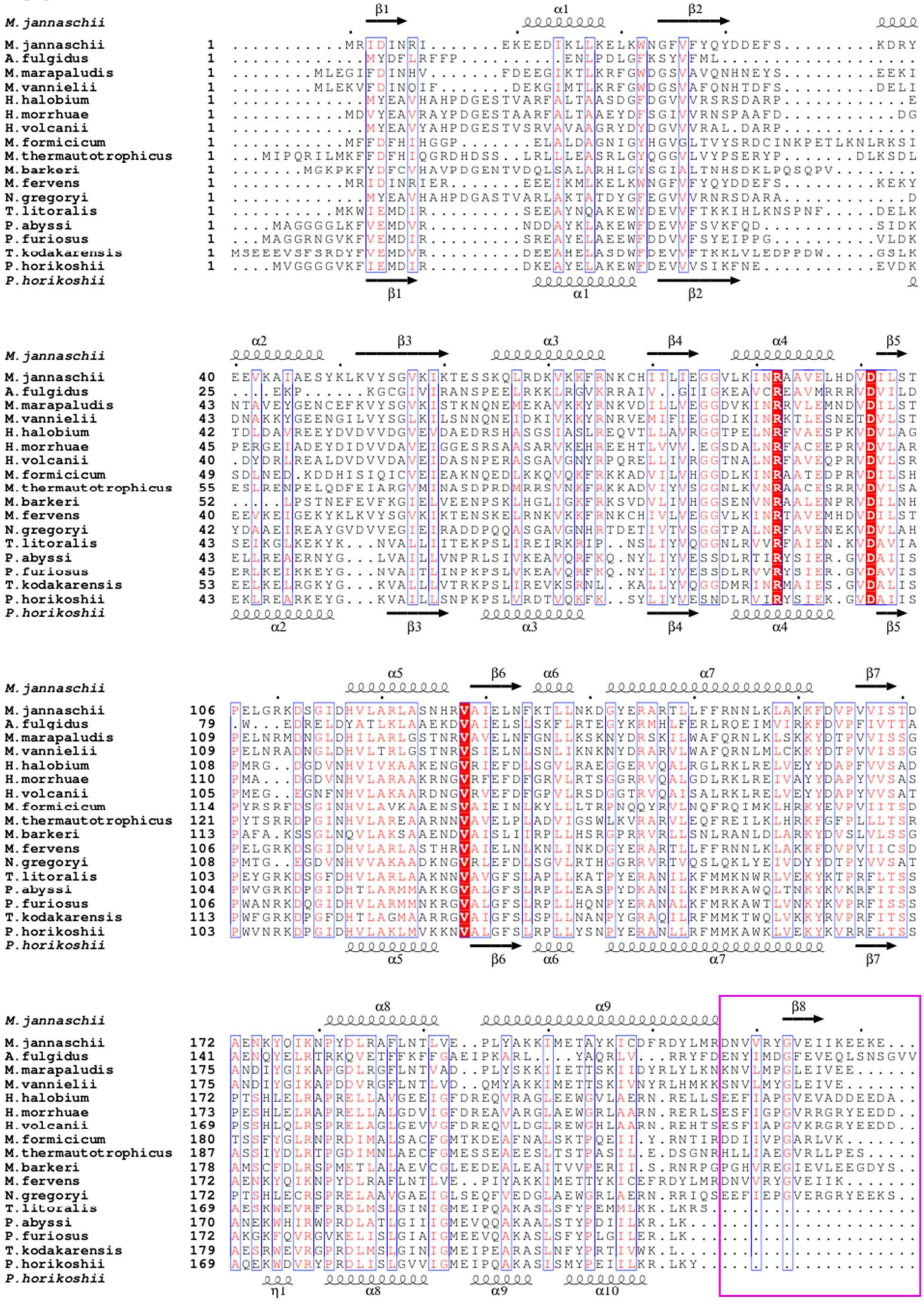

Supplementary Figure 11

Supplementary Fig. 11 Multiple sequence alignment of Rpp30 of A-type and M-type RNase Ps. Sequences of RNase P protein component Rpp30 from 17 representative type-A and type-M archaea including *M. jannaschii* and *P. horikoshii* were aligned. Conserved residues are colored in red. The C-terminal regions of Rpp30 were highlighted with a magenta box. Secondary structure elements were assigned based on the cryo-EM structure of *Mja*RNase P reported in this study and the crystal structures of *P. horikoshii* RNase P protein components.

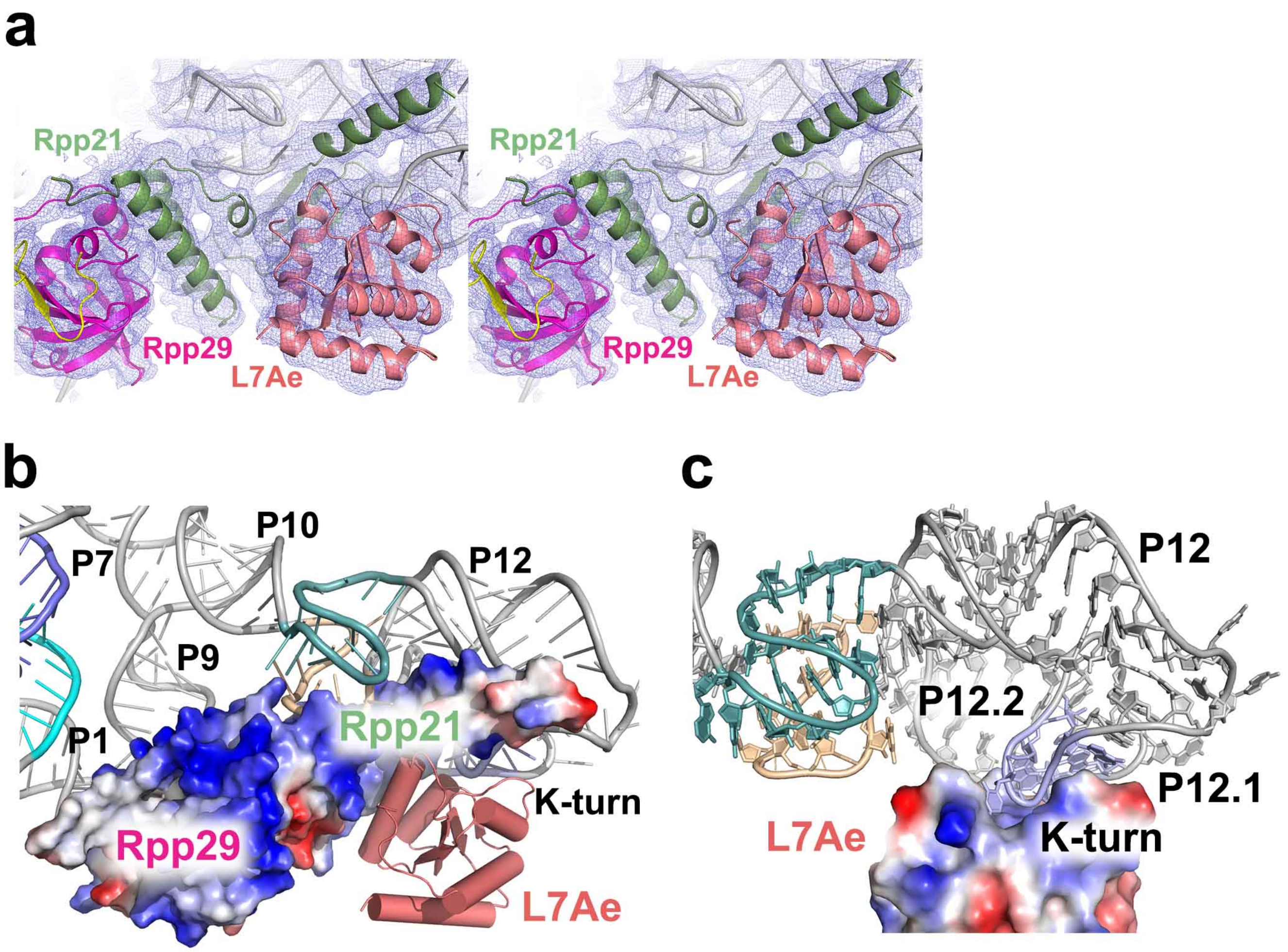

Supplementary Figure 12

Supplementary Fig. 12 Interaction between the Rpp29-Rpp21-L7Ae heterotrimer and RPR. **(a)** Stereo view of the electron density map of the interface between Rpp29-Rpp21-L7Ae and RPR. **(b)** Close-up view of the interface between Rpp29-Rpp21 and the S domain of RPR. **(c)** Close-up view of interface between L7Ae and the K-turn of RPR.

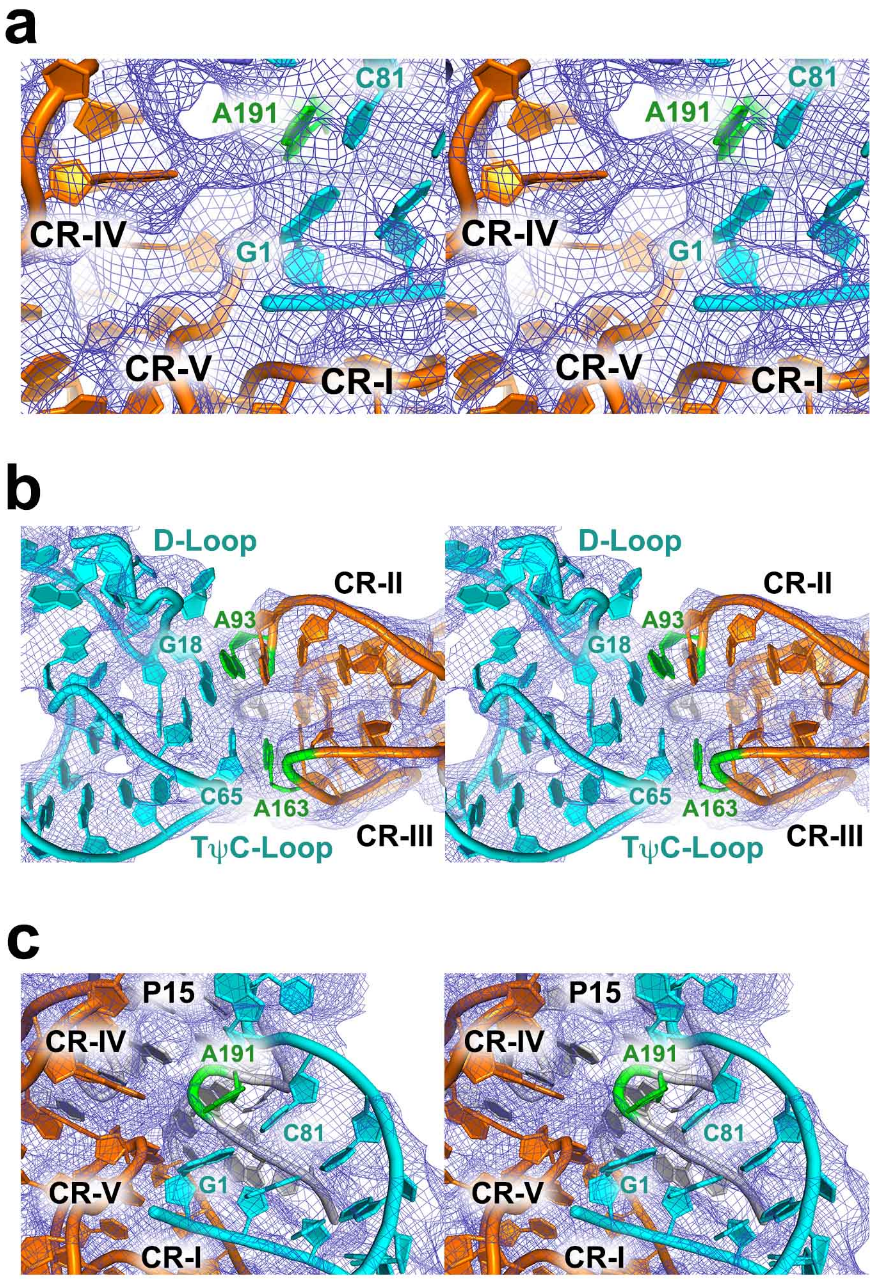

Supplementary Figure 13

Supplementary Fig. 13 Stereo views of the electron density map of the interactions between RPR and tRNA. **(a)** Close-up view of the 5' end of tRNA. CR-I, CR-IV and CR-V of RPR are colored in orange. **(b)** Close-up view of the electron density map of the interface between the TψC and D loops of tRNA and the T-loop anchor in CR-II and CR-III of RPR. **(c)** Stereo view of the electron density map of the central nucleotide A191 (in green) in loop $L_{5\text{-}15}$ of RPR and the first base pair G1-C81 of tRNA.

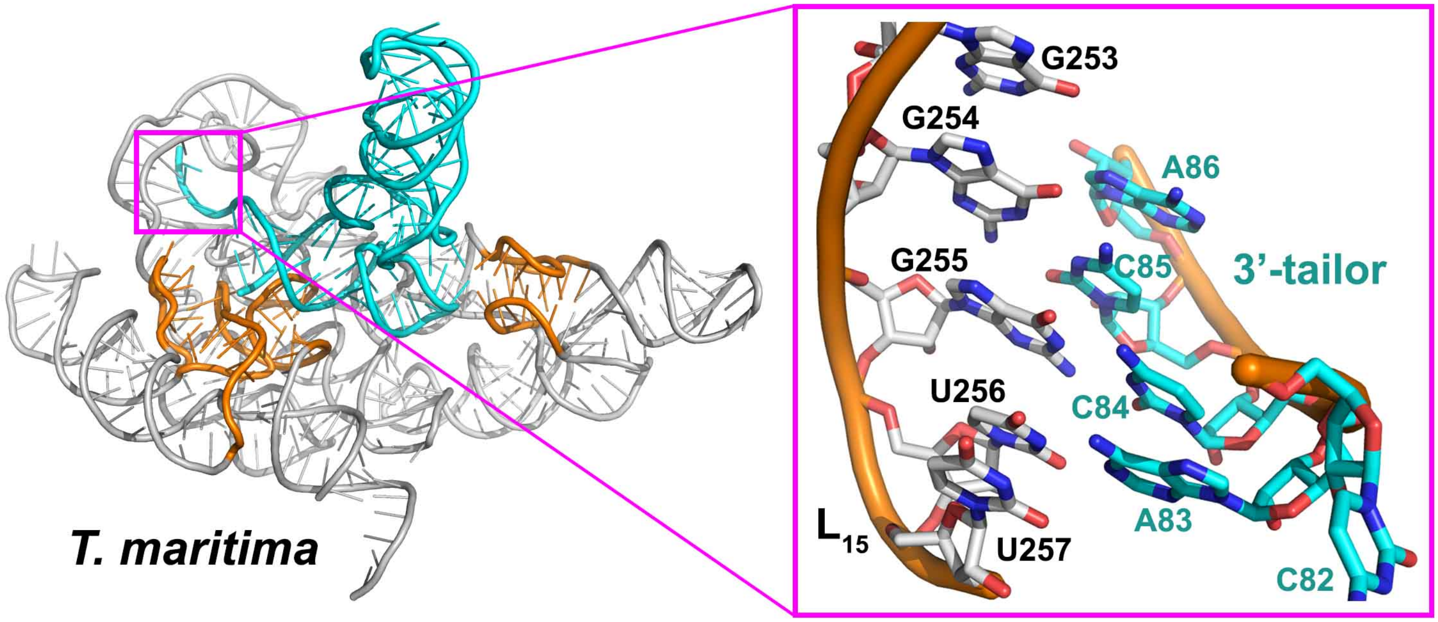

Supplementary Figure 14

Supplementary Fig. 14 Interaction between the 3'-RCCA tail of tRNA and loop L_{15} of *T. maritima* RNase P. Left: Overall view of the interaction between tRNA and *T. maritima* RNase P. Right: Close-up view of the 3'-RCCA tail recognized by base pairing interactions with loop L_{15} of *T. maritima* RNase P.

A type				M type				P type	
P. horikoshii		*H. hispanica*		*M. jannaschii*		*A. fulgidus*		*P. aerophilum*	
tRNA	Sequence	tRNA	Sequence	tRNA	Sequence	tRNA	Sequence	tRNA	Sequence
Ala	ACCA CATA	Ala	ACTC CTGT	Ala	ACCA TTTT	Ala	ATGT TTTT	Ala	ACCA GCCC
Ala	ACCA CAAA	Ala	ATCC TTCT	Ala	ACTA TATA	Ala	ATGC CATA	Ala	ACTA TTTC
Ala	ACCA CGAA	Ala	ACTC CCGT	Arg	GTTA TTTT	Ala	ATTA TTAC	Ala	ACCA CTTT
Arg	GCCA TCAC	Arg	GTGT TCTG	Arg	CTCA TTTA	Arg	GGTA TTTT	Arg	GCCA CTAT
Arg	GCCA GATT	Arg	GTGC AGCG	Arg	GCCA ATTT	Arg	GCTT CTGT	Arg	GCCA TAAT
Arg	GCCA ACTC	Arg	GTTC TCAC	Asn	GCCA TTAA	Arg	ATGG AATT	Asn	GCTG GACT
Arg	GCCA ACAA	Arg	ACTC GATT	Asp	GCCA TTTG	Arg	GTCA GAAA	Asp	GCCA ACCC
Arg	GCCA ACGT	Arg	GTGG CGTC	Cys	TCCA TTTG	Arg	GCTC CAAT	Gln	ACTT CCGC
Asn	GCCA AAGA	Asn	GTCA CTTC	Gln	ACCA TTTT	Asn	GTAT TGTC	Gln	ACCA ATTC
Asp	GCCA TAAT	Asp	GCTT TCTC	Glu	ATCA TTCG	Asp	GCTC TCAT	Glu	ATGA AACA
Cys	TCCA AACT	Cys	TTTT CAGC	Gly	ACCA CTGG	Cys	TCTC TTTA	Gly	ATTG GTCT
Gln	ACCA TTGC	Gln	ACTT CTGG	Gly	ACCA TAAT	Gln	ATCT GTTT	Gly	ACCA CGTT
Gln	ACCA AAAA	Gln	ACTC ACCG	His	CCTT TTTT	Gln	ATGT CTAA	Gly	ATGT CCGC
Glu	ACCA TGTG	Glu	ATTC CAAC	Ile	ACCA TATT	Glu	ACTG CTTT	His	CCCA CGTC
Glu	ACCA GGAA	Glu	ATTT CTTG	Leu	ACCA AATA	Glu	ATTA TCCT	Ile	ACAT CAAG
Gly	ACCA TTAT	Gly	ATCC TTCA	Leu	ACCA TTTT	Gly	ACTG TTTT	Ile	ACCT TGAC
Gly	ACCA CAAC	Gly	ATTT CTCA	Leu	ACCA TAGG	Gly	ATAG CACT	Leu	ACCA CTTC
Gly	ACCA CAAT	Gly	ATTT CTGT	Lys	GCTA TTTC	Gly	ACAT TGGT	Leu	ACCA CCTT
His	CCCA GAAA	His	CCTT TTCG	Met	ACTA TTTC	His	CCTT TAAA	Leu	ACCA CCTC
Ile	ACCA GAAT	Ile	ACTA GCGG	Met	ACCA TATT	Ile	ATTT TTAA	Leu	ACCA GTTT
Leu	ACCA TTTT	Leu	ATCG CCGG	Met	ACCA TTTT	Leu	ATCA CTGA	Leu	ACCA CCTC
Leu	ACCA CTTA	Leu	ATTC GACT	Phe	ACCA TTGA	Leu	ACTG AATT	Lys	GCTA CGAC
Leu	ACCA TGCT	Leu	ATCG CACT	Pro	ACCA TTTG	Leu	ATTT AGTT	Lys	GCCA GTTA
Leu	ACCA ATTC	Leu	ACTC GTTT	Pro	ACCA CTTC	Leu	ATGC TTTT	Met	ATTT TTAT
Leu	ACCA AAAT	Leu	ATAC CACT	Ser	GCTA TCTA	Leu	ATGA TTTA	Met	ACTT TTTT
Lys	GCCA GCCT	Lys	GCTT TCTG	Ser	GCCA CTTC	Lys	GCTG TTTG	Phe	ACCA TCGC
Lys	GCCA AGTT	Lys	GTTC GACC	Ser	GCCA ATGA	Lys	GTAT TTAA	Phro	ATTC TTAA
Met	ACCA TGCT	Met	ACTC CTCT	Thr	TCCA TTTG	Met	ATTT GCCC	Phro	ATTT TATT
Met	ACCA GAAT	Met	ATCC ATTT	Thr	TCCA TTCT	Met	ATGG TTGA	Phro	ATCT ACGT
Met	ACCA TACT	Met	ACTC CTGC	Trp	ACCA TTTT	Met	ATCA GGAA	Ser	GCCT TTCC
Phe	ACCA GAAT	Phe	ATAT TCTG	Tyr	ACCA TCTC	Phe	ATTT CTAT	Ser	GCCT TTTT
Pro	ACCA TTAA	Pro	ACTA CACA	Val	ACCA ATAA	Pro	ACTT TAAA	Ser	GCTT TTCT
Pro	ACCA AAAT	Pro	ACTC AAAA	Val	ACCA TAAT	Pro	ATCA TTGA	Ser	GCCA TATC
Pro	ACCA ATTT	Pro	ATAC TGTT	Val	ACTA TTTT	Pro	ATCT GCTT	Trp	ACCA CTTG
Ser	GCCA CCTG	Ser	GCTT CTCG			Ser	GCTT ACCA	Tyr	ACTA GTCT
Ser	GCCA CACT	Ser	GCCT TTCA			Ser	GCTG AAAC	Val	ACCT TTCC
Ser	GCCA ATCC	Ser	GTTT CTCG			Ser	GCTG TTGT	Val	ATTT AATT
Ser	GCCA GAAT	Ser	GCTT CTCG			Ser	GCTG GGGG		
Thr	TCCA ACAA	Thr	TTCA GGAA			Thr	TTTC CTGA		
Thr	TCCA TTCT	Thr	TTTT CCTG			Thr	TTGC AATA		
Thr	TCCA GCGG	Thr	TTGA TACC			Thr	TTGC TTTA		
Trp	ACCA AGTT	Trp	ATAC CCTT			Trp	ATTT TTAT		
Tyr	ACCA CCAG	Tyr	ATTT CCTC			Tyr	ACTT GAAT		
Val	ACCA TTTC	Val	ATTA CTTC			Val	ATTT TTCT		
Val	ACCA GATT	Val	ATTC ACGC			Val	ATTT ATTT		
Val	ACCA TCGT	Val	ACTC ACGT			Val	ATTT TTGT		

Supplementary Figure 15

Supplementary Fig. 15 3' terminal sequences of tRNAs from representative A-type, M-type and P-type archaea. All of *P. horikoshii* tRNA genes contain the 3'-RCCA sequence. Some *M. jannaschii* and *Pyrobaculum aerophilum* tRNAs do not contain the 3'-RCCA sequence. The 3'-RCCA sequence is highlighted in red.

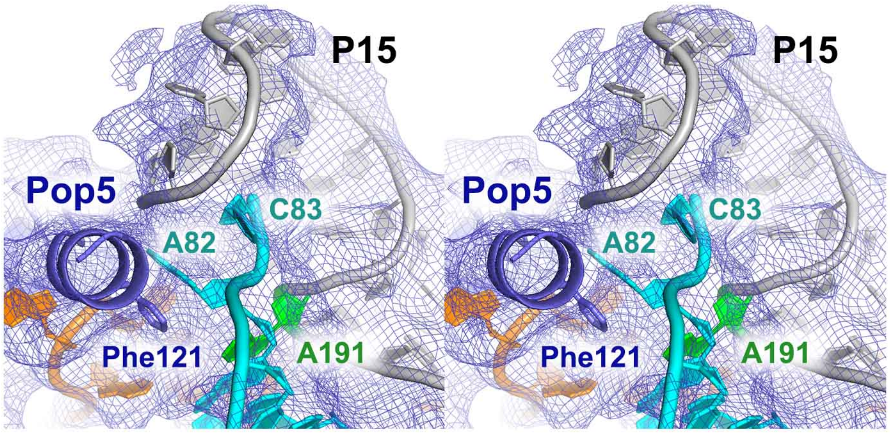

Supplementary Figure 16

Supplementary Fig. 16 Stereo view of the electron density map of the 3' termini of the tRNA substrate. The tRNA is colored in cyan, RPR in gray and Pop5 in slate.

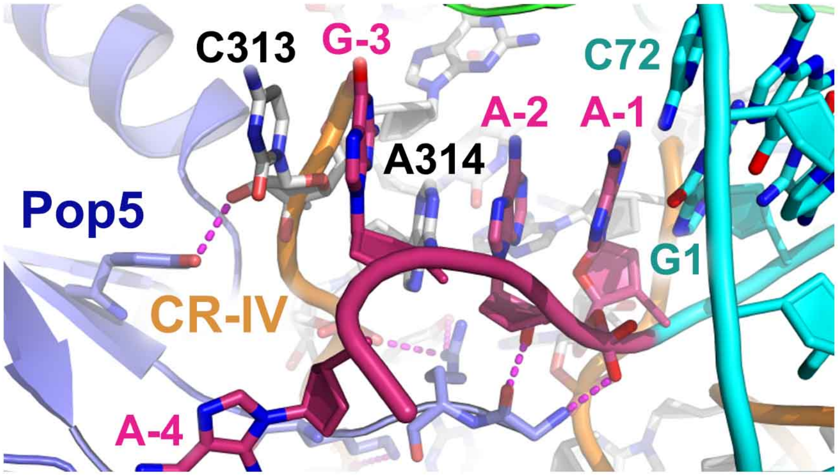

Yeast

Supplementary Figure 17

Supplementary Fig. 17 A close-up view of the interaction between the 5'-leader and RNase P in yeast RNase P-tRNA complex structure. Four nucleotides (A-1, A-2, G-3 and A-4) in the 5′-leader are colored in warmpink. The rest of pre-tRNA and the conserved pseudoknot of Rpr1 RNA are shown in cartoon and colored in cyan and orange, respectively.

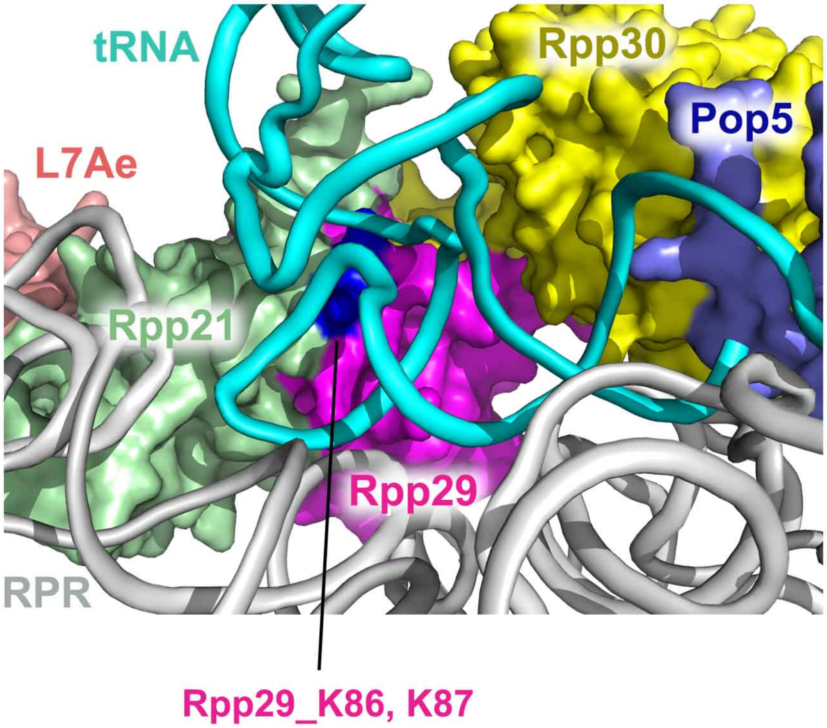

Supplementary Figure 18

Supplementary Fig. 18 Overall view of the interface between the tRNA substrate and protein components Rpp30, Rpp29, and Rpp21. The RNA and protein components are shown in cartoon and surface representations, respectively. Alanine substitutions of *P. horikoshii* Rpp29 Lys121 and Lys122 (equivalent to *M. jannaschii* Rpp29 Lys86 and Lys87) highlighted in blue greatly reduced the enzymatic activity of archaeal RNase P.

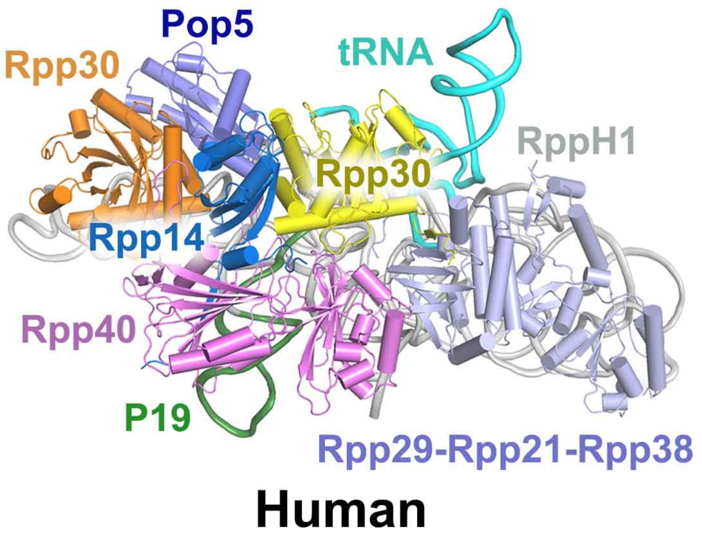

Supplementary Figure 19

Supplementary Fig. 19 Human and yeast RNase P-tRNA complexes adopt a monomeric conformation. Left: In human RNase P-tRNA complex structure, Rpp30 is buttressed by Rpp40 and stem P19 of H1 RNA. Right: In yeast RNase P-tRNA complex, Rpp1 is buttressed by stems P1 and P19 of Rpr1 RNA.

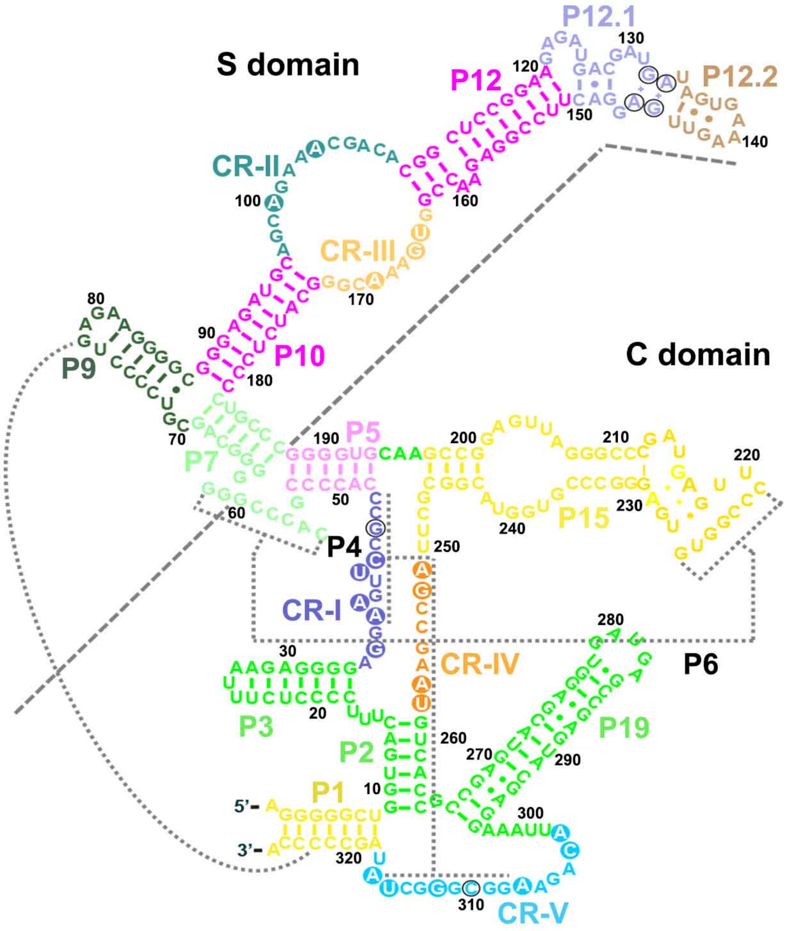

Supplementary Figure 20

Supplementary Fig. 20 Secondary structure of the monomeric mutant RPR. Stems and the conserved regions are colored as in Fig. 3a. To make a monomeric mutant RPR, a P19 stem was inserted in the loop between stems P2 and P4. In addition, a short stem beyond P15 and a linker between stems P5 and P7 were engineered to form a type-A like structure.

Supplementary Table 1. Primary sequences of *M. jannaschii* tRNA genes

tRNA	5' leader	Core region	3' tail
Ala	TACCTAAACTTTGATGATTGATTGTTTTGT	GGGCTGGTAGCTCAGACTGGGAGAGCGCCGCATTGGCTGTGCGGAGGCCGCGGGTTCAAATCCCGCCCAGTCC	ACCA TTTT
Ala	CCAGTCGTGGGCTTGCCTCATAGGGAAAGT	GGGCCCGTAGCTCAGCTGGGAGAGCGCCGGCCTTGCAAGCCGGAGGCCGTGGGTTCAAATCCCACCGGGTCC	ACTA TATA
Arg	AATGGTTGAAATCATGGGATGTAATATAGA	GCCCGGGTCGCCTAGCCAGGATAGGGCGCTGGCCTGCGGAGCCAGTTTTTTCAGGGGTTCAAATCCCCTCCCGGGC	GTTA TTTT
Arg	AAATAAAAATACCATATAATAGCTTATTAA	GCCCTGGTGGTGTAGAGGATATCACGGGGGACTTCGGATCCCCAAACCCGGGTTCAAATCCCGGCCAGGGC	CTCA TTTA
Arg	TTAAAGAGTGCTTACAACCTGAGATAAAGA	GGACCCGTAGCCTAGCCTGGATAGGGCACCGGCCTTCTAAGCCGGGGGTCGGGGGTTCAAATCCCCTCGGGTCC	GCCA ATTT
Asn	TTCAAATCACCCCCGCGGCACCATTGATGT	GCCCCCATAGCTCAGATGGTAGAGCGACGGACTGTTAATCCGTAGGTCGCAGGTTCGAGTCCTGCTGGGGGC	GCCA TTAA
Asp	AATTCTCAAGGTAATCCTTGGCATTACTAT	GCCCTGGTGGTGTAGCCCGGCCTATCATACGGGACTGTCACTCCCGTGACTCGGGTTCAAATCCCGGCCAGGGC	GCCA TTTG
Cys	TTATGTATGCAACTCATAGGTTTTGAAAGA	GCCGGGGTAGTCTAGGGGCTAGGCAGCGGACTGCAGATCCGCCTTACGTGGGTTCAAATCCCACCCCCGGC	TCCA TTTG
Gln	AGTATTTATTAAATTGCTTACGTTTGATGT	AGCCCGGTGGTGTAGTGGCCTATCATCCGGGGCTTTGGACCCCGGGACCGCGGTTCGAATCCGCGCCGGGCT	ACCA TTTT
Glu	TCGGGCCCACCATATTAAAATCACTAATTA	GCTCCGGTGGTGTAGTCCGGCCAATCATGCGGGCCTTTCGAGCCCGCGACCCGGGTTCAAATCCCGGCCGGAGC	ATCA TTCG
Gly	TCTGAGTATGTTATTGTTGCTTGCAATGGA	GCGGCCTTGGTGTAGCCTGGTAACACACGGGCCTGCCACGCCCGGACCCCGGGTTCAAATCCCGGAGGCCGC	ACCA CTGG
Gly	TCTGAGTATGTTATTGTTGCTTGCAATGGA	GCGCCGGTGGTGTAGCCTGGTATCACTCTGGCCTTCCAAGCCAGCGACCCGGGTTCAAATCCCGGCCGGCGC	ACCA TAAT
His	AATCCCTGCCCCTGCACCATAGGTAAAGTG	GCCGGGGTGGGGTAGTGGCCATCCTGGGGGACTGTGGATCCCCTGACCCGGGTTCAATTCCCGGTCCCGGC	CCTT TTTT
Ile	TATTATTTAATTTTTGCTTCTGGTGGTTGT	AGGGCGGTGGCTCAGCCTGGTTAGAGTGCTCGGCTGATAACCGAGTGGTCCGGGGTTCGAATCCCCGCCGCCCT	ACCA TATT
Leu	GAGGTTTATAAATAAAATTTGCCTTTAAGG	GCGGGGGTCGCCAAGCCAGGTCAAAGGCGCCAGATTGAGGGTCTGGTCCCGTAGGGGTTCGCGGGTTCAAATCCCGTCCCCCGC	ACCA AATA
Leu	AGTTCATCTATTCTATAATTCGCTTGCGGT	GCAGGGGTCGCCAAGCCTGGCCAAAGGCGCCGGACTTAAGATCCGGTCCCGTAGGGGTTCGGGGGTTCAAATCCCCTCCCCTGC	ACCA TTTT
Leu	CTATTTTAAGATAATTAAGAATATATATGT	GCAGGGGTCGCCAAGCCTGGCCAAAGGCGCTGGGCCTAGGACCCAGTCCCGTAGGGGTTCCAGGGTTCAAATCCCTGCCCCTGC	ACCA TAGG
Lys	TTGAAACGCGCACGAGCTAAGGGCTGAAGA	GGGCCCGTAGCTCAGTCTGGCAGAGCGCCTGGCTTTTAACCAGGTGGTCGAGGGTTCAAATCCCTTCGGGCCC	GCTA TTTC
Met	TTCGCTTGTTTTGGATTAAAAGTTGAGTGA	AGCGGGGTAGGGTAGCCAGGTCCATCCCGCCGGGCTCATAACCCGGAGATCGGAGGTTCAAATCCTCCCCCCGCT	ACTA TTTC
Met	ATTAATTTTTTATTTTTTTATACCCAATAT	GGGCCCGTAGCTCAGCCTGGTCAGAGCGCTCGGCTCATAACCGAGTGGTCAAGGGTTCAAATCCCTTCGGGCCC	ACCA TATT
Met	TATGGGTTTGTAATTATAATGATGTTGCGT	GCCGAGGTGGCTTAGCTGGTTATAGCGCCCGGCTCATAACCGGGAGGTCGAGGGTTCGAATCCCTCCCTCGGC	ACCA TTTT
Phe	TGTAATGCATAATTATGCAAACATTTGGGT	GCCGCGGTAGTTCAGCCTGGGAGAACGCTGGACTGAAGATCCAGTTGTCGGGTGTTCAAATCACCCCCGCGGC	ACCA TTGA
Pro	TAGATTTTTAAACTTATTATTCTTTTTAAC	GGGGCCGTGGGGTAGCCTGGATATCCTGTGCGCTTGGGGGGCGTGCGACCCGGGTTCAAGTCCCGGCGGCCCC	ACCA TTTG
Pro	CCCCCCCGGGGCTCCATTCTCTTCTTCAGT	GGGCCTGTGGGGTAGCCTGGTCTATCCTTTGGGATTTGGGATCCTGAGACCCCAGTTCAAATCTGGGCAGGCCC	ACCA CTTC
Ser	CCTTATACCCAATTAGTGTTTGCTTTTATT	GCGGGGGTGGCCCAGCCTGGTACGGCGTGGGACTGCTAATCCCATTGGGCAACGCCCAGCCCGGGTTCAAGTCCCGGCCCCCGC	GCTA TCTA
Ser	GAATATATAAAAAATGAAACACGTTTCAGA	GCGGAGGTAGCCTAGCCCGGCCAAGGCGTGGGACTGGAGATCCCATGGGGCTTTGCCCCGCGGGGGTTCAAATCCCCCCCTCCGC	GCCA CTTC
Ser	ATCATTATATTTTTCATATGCCTATTTTGA	GCAGGGGTAGCCAAGCCAGGACTACGGCGCTGGACTTGAGATCCAGTGGGGCTTTGCCCCGCCTGGGTTCAAATCCCAGCCCCTGC	GCCA ATGA
Thr	TAAGATATAGTAATTTAACTATGCTAAGGT	GCCTCGGTAGCTCAGCCTGGCGGAGCGCCTGCTTGGTAAGCAGGAGGTCGCGGGTTCAAACCCCGCCCGAGGC	TCCA TTTG
Thr	ACATCTGTAATTTACTAATACTGCAAACGA	GCCTCGGTGGCTCAGCCTGGTAGAGCGCCTGACTTGTAATCAGGTGGTCGGGGGTTCAAATCCCCCCCGGGGC	TCCA TTCT
Trp	TTTATTCCTGTGAAGATTCTACATTGATGC	GGGGGTGTGGTGTAGCCAGGTCTATCATCGGGGACTCCAGATCCCTGGACCTGGGTTCAAATCCCAGCACCCCC	ACCA TTTT
Tyr	TCAAATCTGGGCAGGCCCACCACTTCGTGT	CCGGCGGTAGTTCAGCCTGGTAGAACGGCGGACTGTAGATCCGCATGTCGCTGGTTCAAATCCGGCCCGCCGG	ACCA TCTC
Val	TATTCAAACATCAGTTAAAACTTATTAGAT	GGGCTCGTGGTCTAGTGGCTATGACGCCGCCCTCACAAGGCGGTGGTCGCGGGTTCGAATCCCGCCGAGCCC	ACCA ATAA
Val	TACACATACTTTAATTTCGCTTGTGGTTGA	GGGCTCGTGGTCTAGATGGCTATGATGCCGCCCTGACACGGCGGTGGTCGGGAGTTCGAATCTCCCCGAGCCC	ACCA TAAT
Val	AGTTAAATTTTTATTGATGCTAAAGGTTGA	GGGCCCATGGTCTAGCTGGCTATGACGTCGCCCTTACAAGGCGAAGGTCGCCGGTTCGAATCCGGCTGGGCCC	ACTA TTTT

Supplementary Table 2. Cryo-EM Data Collection and Refinement Statistics

	*Mja*RNase P	*Mja*RNase P with tRNA
**Data collection**		
EM equipment	FEI Titan Krios	FEI Titan Krios
Voltage (kV)	300	300
Detector	Falcon III	Gatan K2
Pixel size (Å)	1.09	1.32
Electron dose (e-/Å^2)	50	50
Defocus range (μm)	-1.5 ~ -2.5	-1.5 ~ -2.5
**Reconstruction**		
Software	RELION 1.4 and 3.0	RELION 1.4 and 2.1
Number of used particles	84,800	150,000
Symmetry imposed	C2	C2
Final resolution (Å)	4.6	4.3
FSC threshold	0.143	0.143
Map sharpening B-factor (Å^2)	-232	-189
**Model building**		
Software	Coot 0.89	Coot 0.89
**Refinement**		
Software	Phenix 1.14	Phenix 1.14
Map CC	0.600	0.689
**Model composition**		
Protein residues	1,364	1,376
RNA nucleotides	516	688
**Validation**		
R.m.s deviations		
Bond lengths (Å)	0.003	0.003
Bond angles (°)	0.699	0.722
Ramachandran plot (%)		
Preferred region	94.79	93.81
Allowed region	5.21	6.19
Outlier region	0.00	0.00
MolProbity score	1.44	1.73
Clash score (all-atom)	2.91	5.88
Rotamers outliers (%)	0.83	0.66

Uncropped gels in Figure 1a

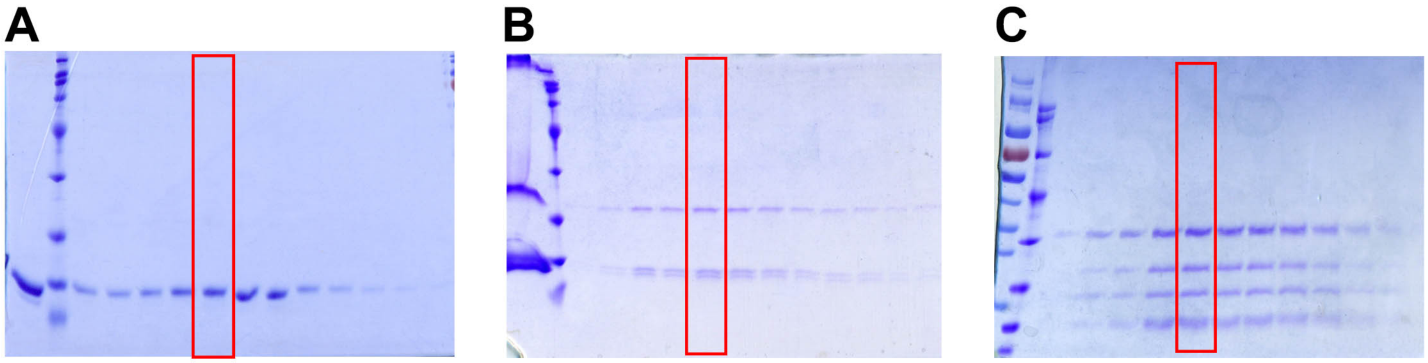

Uncropped gel in Figure 8e

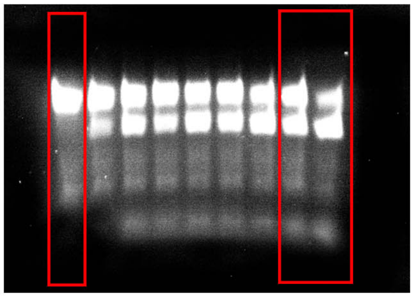

Uncropped gels in Supplementary Fig. 1a

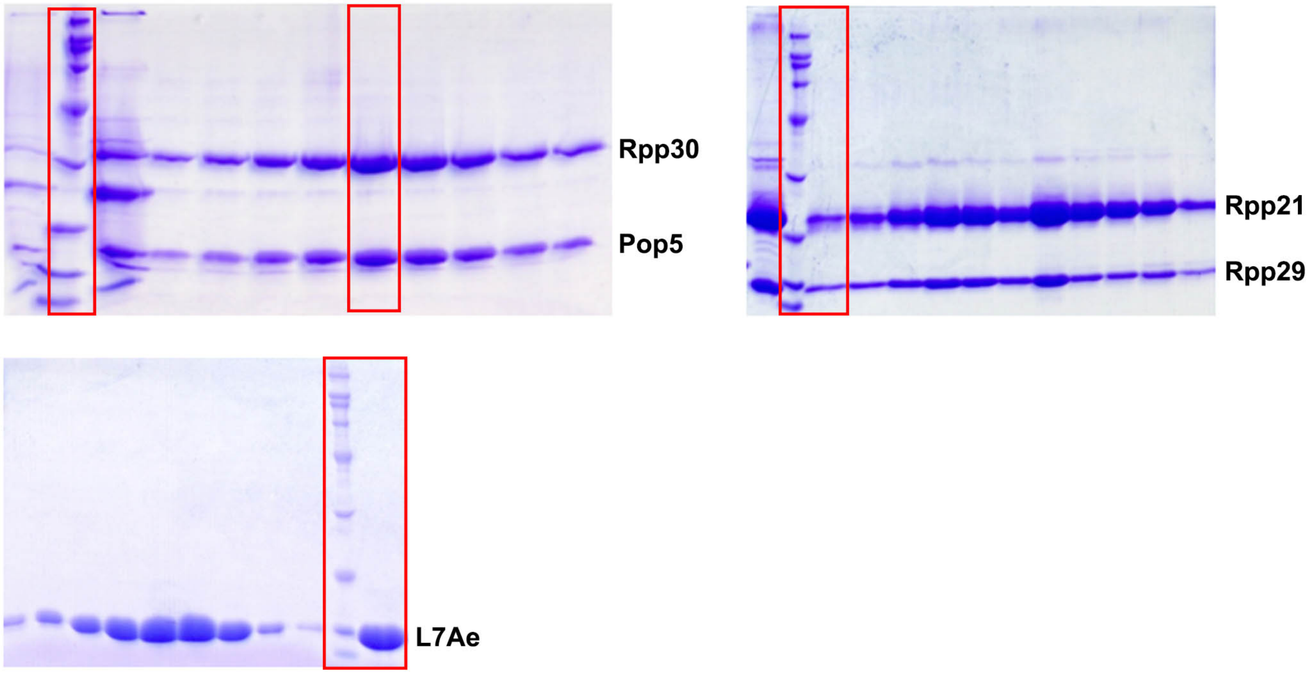

Supplementary Data Set 1
